# Prediction of hallucinogen persisting perception disorder and thought disturbance symptoms following psychedelic use

**DOI:** 10.1093/pnasnexus/pgae560

**Published:** 2025-04-22

**Authors:** Katie Zhou, David de Wied, Robin L Carhart-Harris, Hannes Kettner

**Affiliations:** Centre for Psychedelic Research, Department of Brain Sciences, Imperial College London, London SW7 2AZ, United Kingdom; Centre for Psychedelic Research, Department of Brain Sciences, Imperial College London, London SW7 2AZ, United Kingdom; Centre for Psychedelic Research, Department of Brain Sciences, Imperial College London, London SW7 2AZ, United Kingdom; Department of Neurology, University of California, San Francisco, San Francisco, CA 94158, USA; Centre for Psychedelic Research, Department of Brain Sciences, Imperial College London, London SW7 2AZ, United Kingdom; Department of Neurology, University of California, San Francisco, San Francisco, CA 94158, USA

**Keywords:** psychedelics, HPPD, delusional ideation, magical thinking, adverse effects

## Abstract

Interest in using psychedelic drugs to treat psychiatric disorders is growing rapidly. While modern controlled clinical trials show a favorable safety and efficacy profile, it remains unclear if the risk of side effects would increase with broader use in more heterogeneous populations. To address this, we investigated the frequency and baseline predictors of delusional ideation, magical thinking, and “hallucinogen persisting perception disorder” (HPPD)-related symptoms following psychedelic use in a self-selected naturalistic sample. Using a prospective cohort study, symptoms were assessed in (N=654) participants at one week before a planned psychedelic experience, and at two and four weeks afterward. Across the sample, delusional ideation was found to be reduced one month after psychedelic use (P<0.001) with no changes detected in magical thinking. These findings were in seeming opposition to positive correlations between lifetime psychedelic use at baseline with magical thinking ($r_{s}=0.12$, P=0.003) and delusional ideation ($r_{s}=0.11$, P=0.01), suggesting that schizotypal traits, instead of being caused by, may merely correlate with psychedelic use. Importantly, over 30% of the sample reported HPPD-type effects at the 4-week endpoint, although rarely perceived as distressing (< 1% of the population). Younger age, female gender, history of a psychiatric diagnosis and baseline trait absorption predicted the occurrence of HPPD-like effects. This is in line with prior studies showing a high prevalence of HPPD-like symptoms in psychedelic users, which, however, appear to remain at a subclinical severity in most cases, explaining the comparatively lower prevalence of HPPD diagnoses.

**Significance Statement**

With growing interest in the clinical value of psychedelic drugs, it is crucial to address potential biases favoring their safety and efficacy in controlled modern clinical trials. This prospective cohort study investigated the frequency and prediction of delusional ideation, magical thinking, and hallucinogen persisting perception disorder (HPPD)-type symptoms following psychedelic use in real-world conditions. While aberrant thinking styles either remained unchanged or decreased in the overall sample, one-third (32.1%) of participants reported HPPD-type effects, confirming the importance of assessing such side effects in psychedelic trials. We also show predictors of HPPD-type symptoms, which included younger age, female gender, history of a psychiatric diagnosis, and trait absorption, suggesting that increased caution may be necessary when enrolling younger participants to psychedelic studies.

Following a decades-long human research hiatus, the investigation of the potential that psychedelic-induced altered states of consciousness may possess for aiding the treatment of mental health conditions has seen enormous growth in recent years. Classic psychedelics, such as psilocybin (found in so-called “magic mushrooms”), and lysergic acid diethylamide (LSD), mescaline (found in peyote and san pedro cacti), and dimethyltryptamine (DMT, found in various plants and the amazonian brew ayahuasca), are serotonin 2A-agonists that induce profound alterations in perception, mood, and cognition (1). In addition to promising results for the treatment of mood and addictive disorders (2–4), psychedelic use and psychedelic therapy in particular has been associated with rapid and enduring improvements in mood and wellbeing (2, 5), as well as other behavioral and personality changes (6–11)—including creative thinking (12, 13), nature relatedness (14) and social connectedness (7, 15). Although the use of psychedelics in modern controlled research is generally considered to have a positive safety profile (16, 17), case reports of serious adverse reactions linked to poorly monitored or uncontrolled psychedelic use exist (18–20) supporting the view that use of these substances carries real risk.

In consideration of the possibility that psychedelic substance-assisted treatments may soon be integrated into mental health practice, alongside an ongoing rise in psychedelic use in the general population (21–24), it becomes increasingly pertinent to explore the potential for any long-term, adverse effects of psychedelic use.

Not including adverse reactions driven more distinctly by external factors, such as harm due to accidents, abuse, or coercion by others, the most salient concerns regarding the risks of psychedelic drugs (reviewed e.g. by Schlag et al. (25)) refer to their potential to (i) trigger psychotic episodes (17–19, 26), or more subtly, enhance schizotypal tendencies in healthy individuals (15, 27–29), and (ii) to induce enduring visuoperceptual aberrations, that, when distressing, are clinically recognized as hallucinogen persisting perception disorder (HPPD) (25, 30–33).

HPPD-related phenomena was identified in the literature as early as 1960 (34) and is nowadays considered to include two sub-types. The first, known as HPPD type 1, is defined as “flashbacks”—strictly speaking, not limited to the perceptual domain, these refer to brief (seconds-to-minutes), though full-fledged recurrences of subjective psychedelic effects, including altered perception, cognition, and mood, typically within days to weeks after the initial drug experience. The second type, HPPD type 2 or chronic HPPD, is differentiated from flashbacks based on the persisting nature of its symptoms (35). This, arguably more core domain of HPPD—is typically limited to visuoperceptual aberrations which may wax and wane over months or even years and can include motion “trails,” “halo” effects and intensified colors (21, 23, 24, 36, 37).

Early work with psychedelics recognized their ability to produce transient psychological effects mimicking some important domains of psychosis (38–40), including perceptual changes, bizarre ideation, thought-disorder, and sometimes paranoid or delusional thinking (41). Prolonged adverse effects caused by medical interventions however, appeared to be rare, particularly in controlled settings (34). Analysing data from 44 physicians who administered more than 25,000 doses of lysergic acid diethylamine (LSD) or mescaline to approximately 5,000 subjects between 1950 and 1960, Cohen (34) found an incidence of as few as 10 prolonged psychotic reactions, that is in 0.2% of individuals, leading him to conclude that under controlled conditions, the administration of LSD was generally safe. A similar study of UK clinicians using LSD has found a somewhat higher incidence of 37 prolonged psychoses across 49,000 LSD sessions in 4,300 patients, or 0.9% of subjects (42).

Only into the late 1960s, following the popularization of LSD use, did Cohen and other medical professionals begin to warn against the risks of psychedelic use (43–48). Several commentators have hypothesized that extra-pharmacological or contextual factors may also impact the likelihood of experiencing prolonged adverse reactions, such as psychotic episodes or HPPD (43–48), with recent supportive evidence for this here (49).

To investigate these hypotheses, observational studies of real-world psychedelic use are essential, considering that rigorous screening procedures in modern controlled trials tend to exclude subjects considered at-risk for adverse outcomes (2, 3). Indeed, analysis of the long-term effects of psilocybin in 110 healthy subjects, using pooled experimental data, found no persisting perception disorders, prolonged psychosis, or other long-term impairment of functioning in any of the subjects, although one case of short-lived psychological iatrogenesis was described (50). Similarly, until one recent multisite trial in treatment-resistant depression (51), only a small number of serious adverse events had been reported in modern clinical studies of serotonergic psychedelics for depression (2, 52), end of life anxiety (53, 54), or substance use disorders (55), including no cases of HPPD (56).

In comparison, cross-sectional survey studies of naturalistic psychedelic users have found incidences of visual abnormalities lingering post-psychedelic use ranging from 11% (2) to as high as 60%, although far fewer (4%) experienced these symptoms as distressing, a necessary criterion for diagnosing HPPD (56). To our knowledge, no representative data exist that could inform on the prevalence of prolonged psychotic reactions following naturalistic psychedelic use, although large-scale population studies finding lowered rates of psychotic symptoms in psychedelic users, compared with nonpsychedelic users, suggest that this frequency is unlikely to be high (57). Moreover, one recent study of ours which specifically sought to recruit such cases, also supported their low prevalence and context-sensitivity, when they do indeed occur (49).

The present study sought to investigate changes in delusional and magical ideation before vs. after psychedelic use, using online naturalistic prospective sampling. We also assessed the prevalence, and predictors of HPPD-type effects. Data were collected through a specially designed prospective online survey recruiting individuals who were planning to take a psychedelic through their own initiative. Measures were recorded at multiple time points before and after the psychedelic experience. It was hypothesized that baseline levels of delusional ideation and magical thinking would positively correlate with lifetime psychedelic use. Exploratory regression models were conducted to assess whether demographics, personality trait absorption, drug use history, and details of use during the acute experience, would predict the occurrence of HPPD-type effects and changes in delusional and magical ideation.

## Results

### Demographics

The following sample sizes were collected for each of the surveys at baseline (Table 1), 2 weeks post-acute experience, and 4 weeks post-acute experience: N=654, N=315, N=212. The average age was 28.9 years (SD=10.5). The majority of the population was male (n=485, 74.2%), university educated or above (n=504, 77.1%), and around half were employed (n=337, 51.2%), with the rest comprised of students (n=256, 39.1%). Just over a third of the cohort were “novice” users (five or fewer psychedelic experiences, n=250, 38.2%) with the remaining two-thirds “experienced” (six or more past experiences, n=404, 61.8%). Nearly, a third reported having ever been diagnosed with at least one psychiatric condition (33.0%). Just over half the cohort reported taking LSD/1P-LSD (n=143, 54.0%) during the acute experience, followed by Psilocybin/magic mushrooms/truffles (n=108, 40.8%) (Table 2). Only a minority of the cohort had an acute experience in a retreat setting (n=67, 17.7%) (Table 2).

**Table 1.** Demographic characteristics at baseline.

	Novice	Experienced	Total
	250 (38.2%)	404 (61.7%)	654 (100%)
*Demographic characteristics*			
*Age*	27.0±10.3	30.1±10.4	28.9±10.4
*Gender*			
Male	181 (72.4%)	304 (75.2%)	485 (74.2%)
Female	69 (2.4%)	96 (23.7%)	165 (25.2%)
Other	–	4 (1%)	4 (0.6%)
*Psychiatric history*			
Major depressive disorder	67 (27%)	28 (6.9%)	95 (14.5%)
Bipolar disorder	9 (3.6%)	10 (2.5%)	19 (2.9%)
Schizophrenia	1 (0.4%)	0 (0%)	1 (0.2%)
Anxiety disorder	80 (32%)	29 (7.2%)	109 (16.7%)
Substance abuse disorder	7 (2.8%)	14 (3.5%)	21 (3.2%)
Hallucinogen persisting	2 (0.8%)	3 (0.7%)	5 (0.8%)
perception disorder	11 (4.4%)	5 (1.2%)	16 (2.4%)
Psychotic disorder	1 (0.4%)	1 (0.2%)	2 (0.3%)
ADHD	36 (14.4%)	15 (3.7%)	51 (7.8%)
Obsessive compulsive	7 (2.8%)	4 (1.0%)	11 (1.7%)
disorder			
Alcohol dependence	4 (1.6%)	6 (1.5%)	10 (1.5%)
Eating disorder	3 (1.2%)	3 (0.7%)	6 (0.9%)
*Psychedelic use* ^a^			
Never	–	–	–
Not within the last 6 months	3 (1.5%)	3 (1.0%)	6 (1.0%)
Once	100 (50.8%)	89 (22.0%)	189 (31.4%)
2–5 times	90 (45.7%)	182 (45.0%)	272 (45.3%)
6–10 times	3 (1.5%)	81 (20.0%)	84 (13.8%)
11–20 times	–	33 (8.2%)	33 (5.5%)
21–50 times	–	10 (2.5%)	10 (1.7%)
51–100 times	–	5 (1.2%)	5 (1.0%)
More than 100 times	1 (1%)	1 (< 1.0%)	2 (< 1%)

${\;}^{a}$ During the last 6 months.

**Table 2.** Characteristics of the acute psychedelic experience.

	Novice${\;}^{a}$	Experienced${\;}^{a}$	Total${\;}^{a}$
	108 (40.8%)	157 (59.2%)	265 (100%)
*Psychedelic drug type*			
LSD/1P-LSD	61 (56.5%)	82 (52.2%)	143 (54.0%)
Psilocybin/magic mushrooms/truffles	46 (42.6%)	62 (39.5%)	108 (40.8%)
DMT/5-MeO-DMT	5 (4.6%)	7 (1.7%)	12 (4.5%)
Ayahuasca	19 (17.6%)	24 (4.5%)	43 (16.2%)
Mescaline (Peyote, San Pedro)	3 (2.8%)	5 (3.2%)	8 (3.0%)
Iboga/ibogaine	0 (0%)	1 (0.6%)	1 (0.4%)
2C-B	2 (1.9%)	1 (0.6%)	3 (1.1%)
Other	10 (9.3%)	7 (4.5%)	17 (6.4%)
*Retreat setting*	37 (34.3%)	24 (15.3%)	67 (25.3%)

${\;}^{a}$ Of those who answered the questions on setting and drug type taken during the acute experience.

### Delusional ideation and magical thinking relative to lifetime psychedelic use

**Fig. 1.**
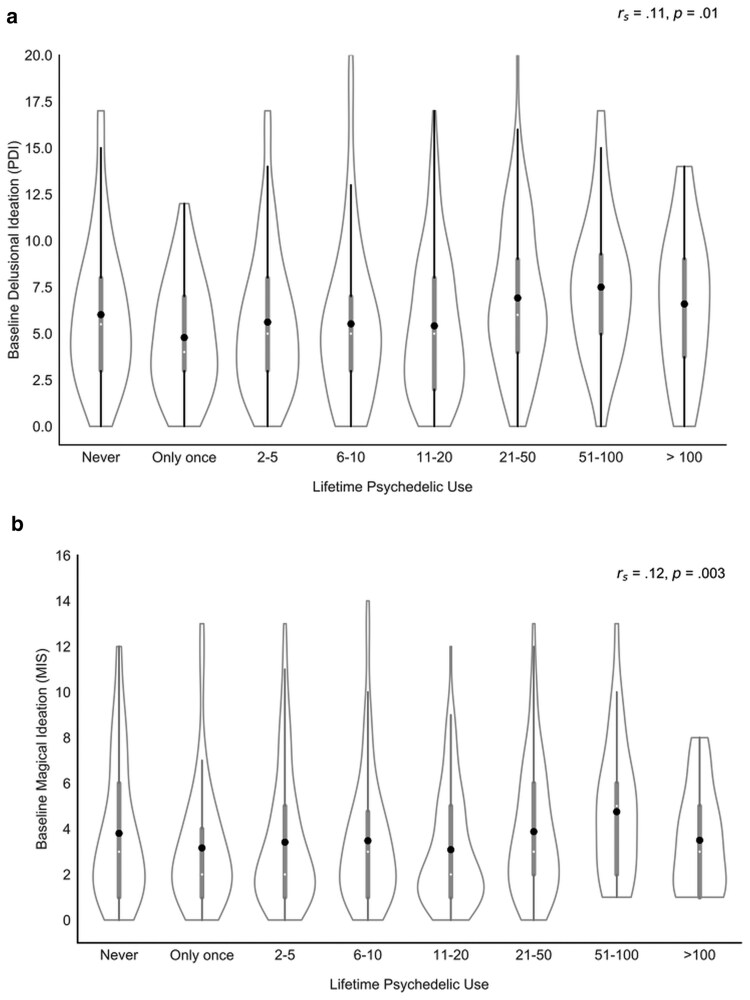
Distribution of a) delusional ideation (PDI score) and b) magical ideation (MIS score) across lifetime psychedelic use. Dots representing the mean.

There was a weak, but significant positive correlation between lifetime psychedelic use and baseline delusional ideation ($r_{s}=0.11$, P=0.01; Fig. 1a). There was also a weak, but significant, positive correlation between lifetime psychedelic use and baseline magical ideation ($r_{s}=0.12$, P=0.003; Fig. 1b). These persisted when correcting for age (Peters’ Delusions Inventory [PDI]: $r_{s}=0.11$, P=0.004. Magical Ideation Scale [MIS]: $r_{s}=0.13$, P=0.001; Fig. 1). Exploring the “distress” component of the PDI, there was no correlation with lifetime psychedelic use ($r_{s}=0.01$, P=0.06).

### Longitudinal changes in delusional ideation and magical thinking

**Fig. 2.**
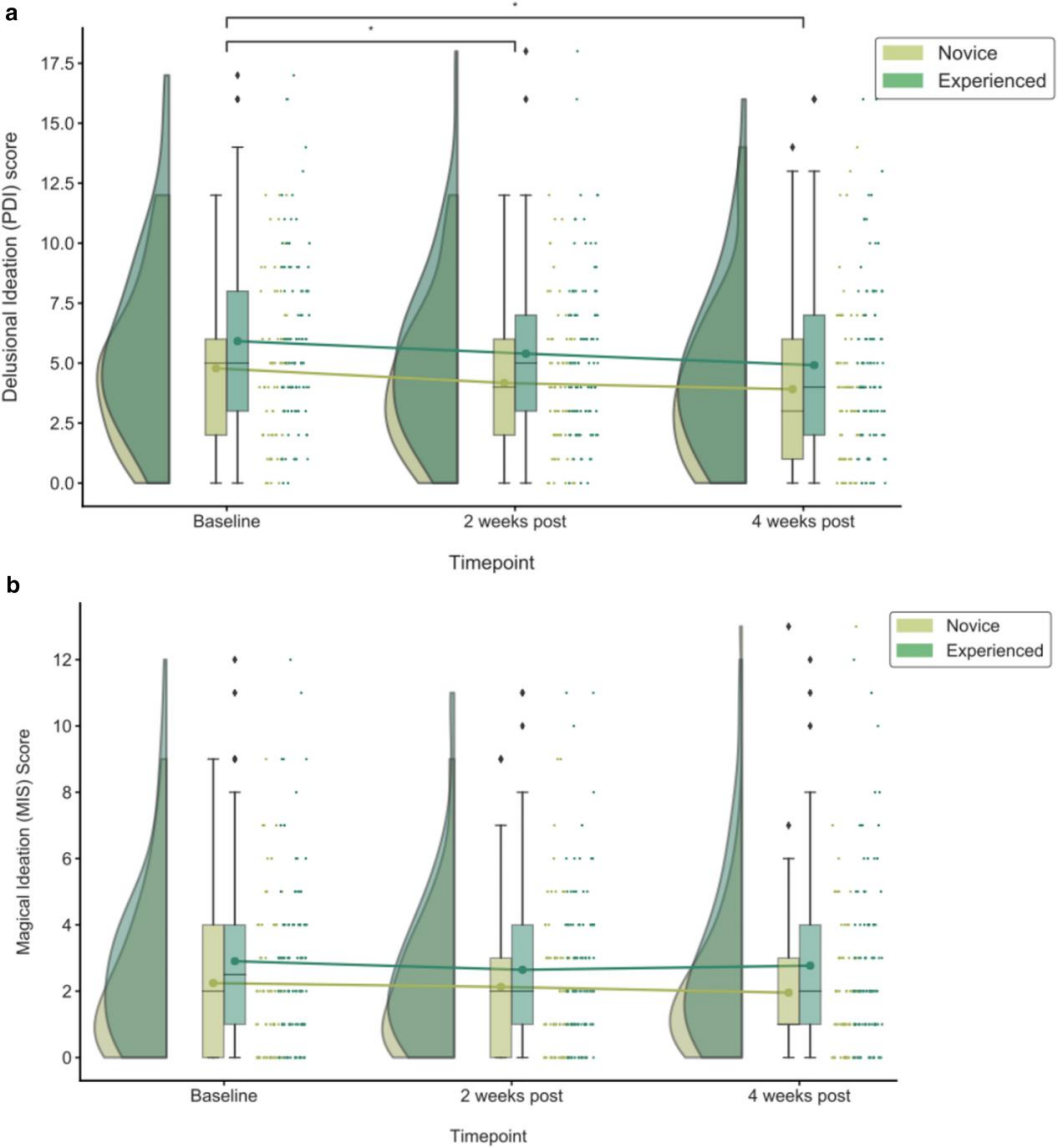
Raincloud plot showing raw data, boxplots and mean with 95% CI for changes in a) delusional ideation (PDI score) and b) magical ideation (MIS score) following the psychedelic experience in novice (lifetime psychedelic use < 6 times) and experienced (lifetime psychedelic use > 5 times) psychedelic users. Dots representing the mean. ${\;}^{*}$  P<0.05, cohort as a whole.

The study population as a whole, demonstrated a statistically significant reduction in delusional ideation after the acute psychedelic experience ($\chi^{2}=15.47$, P≤0.001; Fig. 2a). The decrease in delusional ideation from baseline to 2 weeks (Z=−3.60, P≤0.001), persisted to 4 weeks (Z=−5.19, P≤0.001; Fig. 2a) after the psychedelic experience. Novice users (< 6 lifetime psychedelic experiences), exhibited significant reductions in delusional ideation from baseline (M=5.89, SD=3.87) to 2 weeks after the acute experience (Z=−2.59, P=0.010), and 4 weeks (M=3.91, SD=3.21; Z=−3.31, P≤0.001) after the experience. There were no significant changes in magical ideation from baseline to 4 weeks after the acute experience (Fig. 2b) within experienced users (> 5 lifetime psychedelic experiences).

### Predictors of increased delusional ideation and magical thinking

Slightly less than a quarter (23.8%) of participants showed increased delusional ideation at the 4-week endpoint, with a mean change of 1.8 points. A logistic regression model to investigate persistent increases in delusional ideation at 4 weeks did not find any significant predictors (SI appendix Table S1). However, the odds for reporting increased magical ideation, which occurred in 28.1% of participants was found to increase by 6.0% (95% CI [0.98, 1.03], P<0.001) for a one-unit increment in baseline personality trait absorption (Table 3).

**Table 3.** Results from the logistic regression model investigating predictors of magical ideation (MI).

Variable	Coefficient	Std. error	*z*	*P*-value	OR [95% CI]
Age	−0.03	0.02	−1.27	0.20	0.99 [0.96, 1.03]
Gender	0.66	0.51	1.28	0.20	0.66 [0.29, 1.50]
**Absorption**	**0.06**	**0.02**	**4.17**	**< 0.001**	**1.01 [0.98, 1.03]**
LSD use${\;}^{a}$	−0.40	0.49	−0.81	0.42	1.15 [0.48, 2.76]
Dose	−0.16	0.19	0.41	0.41	0.80 [0.56, 1.14]
Setting	0.48	0.86	0.56	0.57	0.81 [0.38, 3.67]
Polydrug use${\;}^{a}$	0.39	0.48	0.81	0.42	1.26 [0.55, 2.88]
Use of psychedelic drugs${\;}^{b}$	−0.17	0.20	−0.85	0.39	0.73 [0.48, 1.10]

${\;}^{a}$ During the acute experience. ${\;}^{b}$During the last 6 months.

Significant predictor(s) in bold.

### HPPD-type effects

One-third of the 212 individuals who continued to the 4-week endpoint reported at least one HPPD-like effect (n=68, 32.1%) (Table 4), with approximately half of this subgroup reporting two or more effects (n=35). The most frequently reported HPPD-related symptoms were “intensified colors” (n=29, 18.6%) and “positive afterimages” (n=28, 17.9%). Visual distortions such as “macropsia” (n=6, 3.8%) and “micropsia” (n=5, 3.2%) were the least common. There was no significant difference in the likelihood of reporting HPPD effects between novice and experienced participants (P=0.64). Amongst the 68 users reporting at least 1 HPPD-related effect, only 2 subjects (< 1% of the total sample, or 2.9% of those who reported any HPPD-type effects) expressed that these effects were distressing to them. Both subjects also confirmed that these effects were not due to any preexisting medical condition, but were rather perceived to be a consequence of their psychedelic use. One of these individuals endorsed experiencing all nine HPPD-type effects listed in the questionnaire, compared with the other who reported only one effect, namely, “false perceptions of movement in peripheral visual fields.” Both were experienced users, claiming to have used psychedelics more than 10 times. One other individual also reported all nine effects but did not report them to be distressing.

**Table 4.** Frequency of each HPPD-type symptom.

HPPD Symptom	*n*	%
Geometric hallucinations	19	12.2
Flashes of color	12	7.7
False perceptions of movement in peripheral visual fields	26	16.7
Intensified colors	29	18.6
Trails of images of moving objects	17	10.9
Positive afterimages	28	17.9
Halos around objects	14	8.9
Macropsia	6	3.8
Micropsia	5	3.2

### Predictors of HPPD-type effects

A logistic regression model was performed to analyze relationships between HPPD-related symptomatology and age, gender, psychiatric history (yes/no), baseline personality trait absorption and the frequency of psychedelic use during the last 6 months, plus aspects of the acute experience, including setting (retreat vs nonretreat), drug dose, drug type (LSD vs. other psychedelic), and nonpsychedelic drug use (i.e. polydrug use) (Table 5). It was found that, holding all other predictor variables constant, the odds of HPPD-type effects occurring decreased by 63.4% (95% CI [−1.91 , −0.10]) for males compared with females, decreased by 6.2% (95% CI [−0.10 , −0.02]) with each unit increment in age, and increased by 202% (95% CI [1.20, 6.37]) for 3.4% (95% CI [0.001, 0,06]) for each unit increment in baseline personality trait absorption.

**Table 5.** Results from the logistic regression model performed investigating for predictors of HPPD-type symptoms.

Variable	Coefficient	Std. error	*z*	*P*-value	OR [95% CI]
**Age**	**−0.06**	**0.02**	**−3.19**	**< 0.001**	**0.94 [0.90, 0.97]**
**Gender**	**−1.00**	**0.46**	**−2.17**	**0.03**	**0.37 [0.15, 0.91]**
**Absorption**	**0.03**	**0.01**	**2.57**	**0.01**	**1.03 [1.01, 1.06]**
**Psychiatric history**	**1.11**	**0.47**	**2.35**	**0.02**	**3.02 [1.20, 6.37]**
LSD use${\;}^{a}$	0.49	0.48	1.02	0.31	1.63 [0.59, 2.43]
Dose	−0.29	0.18	−1.59	0.11	0.75 [0.51, 1.25]
Setting	−1.18	0.84	−1.41	0.16	0.31 [0.06, 1.15]
Polydrug use${\;}^{a}$	0.62	0.46	1.35	0.18	1.85 [0.77, 3.13]
Use of psychedelic drugs${\;}^{b}$	0.02	0.23	0.08	0.94	1.02 [0.65, 1.63]

${\;}^{a}$ During the acute experience. ${\;}^{b}$During the last 6 months.

Significant predictor(s) in bold.

It was found that, holding all other predictor variables constant, the odds of HPPD-type effects occurring decreased by 63.4% (95% CI [0.15, 0.91]) for males compared with females and decreased by 6.2% (95% CI [0.90, 0.97]) with each unit increment in age. The odds of HPPD-type effects occurring increased by 3.4% (95% CI [1.01, 1.06]) for each unit increment in baseline personality trait absorption. Additionally, history of a psychiatric diagnosis significantly increased the odds of at least one HPPD-type effect by 202.0% (95% CI [1.20, 6.37]). No specific psychiatric diagnosis was found to significantly predict HPPD-type effects (SI appendix Table S2).

## Discussion

The present study sought to investigate potentially undesirable psychological outcomes occurring within a 4-week period of an acute psychedelic experience. Specifically, we assessed delusional thinking and magical ideation, and persisting visual alterations corresponding to HPPD-type effects.

### Delusional ideation and magical thinking

Directionally contradicting a prior hypothesis of ours, we observed a decrease in delusional ideation after (vs. prior to) a psychedelic experience. This result emphasizes caution when inferring a causal connection between observations of elevated schizotypal traits among individuals with greater lifetime psychedelic use, i.e. the association, while real, may not have been caused via psychedelic use. There are a few possible explanations for this apparent paradox. One is that the relationship between lifetime psychedelic use and trait schizotypy is driven by other underlying factors. Greater impulsivity and risk-taking tendencies—coincident with higher trait schizotypy (58, 59)—may draw certain individuals towards sampling psychedelics and their novel effects (60). Additionally, latent or manifest pathology might be a factor, motivating certain individuals, including those with higher schizotypal traits, to seek self-medication via psychedelics. Another possibility is that the decreases in schizotypal thinking observed within 4 weeks of psychedelic use were temporary or use-dependent. For example, schizotypal thinking may rebound after an initial decrease—perhaps due to a precarious short-lived impact on mental health—i.e. a so-called “honeymoon” or “too good to be true” type phenomenon. Additionally, while well-intentioned psychedelic use could improve mental health outcomes (5, 61), repeated use (or abuse) of psychedelics could flip the relationship in an overly iatrogenic direction—i.e. reflecting an “inverse U” type relationship between psychedelic use and mental health outcomes. Future work is required to examine whether trait schizotypy is especially vulnerable to such a phenomenon.

The average delusional ideation score of our sample lies within the range of mean PDI scores found in other nonpsychiatric populations (62–65). In the present sample, experienced users reported higher scores than novice users, on average endorsing one more item in the scoring system. For reference, “deluded or psychotic populations” score much higher, ranging from 7.4 (66) to 11.9 (63).

Baseline magical ideation in the present self-selected sample was nearly twice as high as scores reported in general populations (67), suggesting a skew toward magical ideation in psychedelic users. In line with this, the frequency of lifetime psychedelic use at baseline was found to correlate with higher baseline levels of magical ideation. While this relationship does not entail causality, we are reminded of previous findings of group-level shifts in metaphysical beliefs including supernatural beliefs following psychedelic use both naturalistically and in a clinical trial (68) that could be viewed as suggestive of psychedelic use itself being causal in driving increases in magical ideation.

Among predictors of post-psychedelic use changes in schizotypal traits, baseline absorption was found to be a strong predictor of magical ideation, which was unsurprising considering a component of the MODTAS scale of absorption is defined by “proneness to imaginative states.” Moreover, absorption itself, by-definition, refers to a propensity to become absorbed or immersed in mental imagery, particularly fantasy (69). Although no significant predictors for increased delusional ideation were found, future work should further investigate other aspects such as drug type or personality traits to determine an at-risk profile.

We advise caution on interpreting mild-to-moderate scores on absorption, delusional ideation (without distress), or magical ideation as reflecting pathology. It is possible that such phenotypes could confer certain adaptive advantages, such as enhanced divergent thinking and related creative problem solving (70).

### HPPD-type effects

HPPD-related effects were endorsed by close to a third of participants (32.1%), although only two subjects (i.e. < 1%) perceived these to be distressing, thus disqualifying a HPPD diagnosis for 97% of those who reported HPPD-related effects. This pattern of high rates of HPPD-related effects but low rates of actual HPPD (i.e. lacking the crucial distress component) is consistent with prior studies on HPPD frequency (35, 71–73).

While the DSM-V proposes a prevalence of HPPD in 4–4.5% of psychedelic drug users (American Psychiatric Association, 2013), a questionnaire-based study on the website erowid.org found that as many as 61.7% of respondents reported having had unusual visual experiences lingering on at least one occasion after psychedelic drug use (73). In another previous online survey, which specifically assessed visual effects in response to psychedelics, 40% endorsed persistent changes in visual perception (72), closer to the present findings of 32%.

Importantly, however, the proportion of people reporting distressing HPPD-type effects is remarkably convergent across studies, at 3–4% of those who report any visual aftereffects of psychedelic use (present study and (72, 73)). Across these studies, the frequency of HPPD among psychedelic users overall, would thus be estimated at < 1%, which is at least four times lower than the estimate provided by the DSM-V. This suggests that the DSM-V reported prevalence should be revised to better reflect existing and emerging data. The point to emphasize strongly, is that for most users, HPPD-related aftereffects are not considered distressing.

To our knowledge, no cases of HPPD have been reported in participants of clinical trials of psychedelics (2, 52, 56, 74)—even when formally probed using a similarly structured (DSM-V-consistent) method, as used in the trial of psilocybin vs. escitalopram for depression (3).

Offering speculative explanations for this apparent discrepancy between naturalistic data for and psychedelic therapy, it is possible that yet uninvestigated factors, such as the use of eyeshades and closed-eye experiences may be protective of persistent visual disturbances. Conceivably, the visual system might be subject to greater stress in the context of open-eye visual hallucination where drug-induced anomalous visual experiences need to be reconciled with visual input from the environment. From a predictive coding perspective, altered visual perception induced by the drug could update perceptual priors—and this effect could be more likely with eyes-open as prediction errors will occur due to input-to-inference mismatch. Future studies on psychedelic effects in humans may benefit from assessing setting in a more structured way, including time spent by participants with open eyes, as opposed to closed eyes, or wearing eyeshades.

The finding of trait absorption being linked to HPPD-type symptoms is unsurprising considering its association with hallucination-proneness (75) and recent work linking trait absorption to HPPD in a retrospective sample (35, 76, 77). Whilst in this context, greater trait absorption might potentially seem undesirable, it is important to consider that a propensity to become deeply immersed in stimuli—as displayed by individuals with high absorption—may confer certain psychological advantages (78, 79) and has also been found to be predictive of some of the acute subjective effects that are known to render psychedelic experiences therapeutically meaningful (80–82).

We also found that younger age was associated with a greater likelihood of reporting HPPD-related effects. This suggests that the role of age and critical windows for perceptual learning should be considered when investigating psychedelic effects in humans. Adolescence represents the peak age of onset for mental disorders (83) and the adolescent brain is known to be particularly vulnerable to the negative consequences of substance use (84). Heightened neuroplasticity during adolescence might thus explain the higher likelihood for younger people in the current sample to report HPPD-type effects following psychedelic use, which should be taken into account when defining minimum recruitment age in future studies using psychedelics. Limiting this conclusion, it is possible that younger participants could have used psychedelics within settings involving greater sensory stimulation or polydrug use (such as at nightclubs or parties) (85), or other stressors, which may have increased the likelihood of experiencing persistent perceptual effects—confounders that should be controlled for in future observational studies. There is also the possibility that they may have taken the psychedelic with less education and therapeutic intention, and in higher-than-average doses.

The observed higher likelihood of HPPD-type effects in female participants was surprising, considering that gender has not previously been identified as a predictor of response to psilocybin (80, 86, 87). Sexually dimorphic differences in drug pharmacokinetics may account for the effect, with studies finding pharmacokinetics to strongly predict adverse drug effects in females (88), albeit not in a manner exclusive to psychedelics, and neither are there clear sex differences in serotonin 2A receptor functioning (89). The lower-than-average weight of females (90) will affect dose to weight ratio—potentially conferring more intense responses in females with the same dose, although BMI has not been found to predict the intensity of subjective effects with psychedelics (91). Menstrual-cycle-related hormonal and biochemical effects may be investigated in the future to further examine any sex-differences.

The finding that a history of a psychiatric diagnosis was associated with a greater odds of experiencing HPPD-type effects was expected. This is in line with existing literature, which has consistently found more pronounced neurobiological responses to psychedelics in individuals with psychiatric conditions (52, 92), and greater susceptibility to perceptual disturbances (7, 52, 92, 93). These results highlight the importance of carefully screening for mental health conditions and stress the need for enhanced support such as continuous monitoring and comprehensive aftercare, to mitigate the risk of persistent visual disturbances and other adverse effects.

While LSD use is often associated with various perceptual disturbances (94–96), it did not emerge as a key predictor of HPPD-type symptoms. This implies that other factors, such as age, gender, or preexisting mental health conditions, may play a more critical role in determining the susceptibility to HPPD-type effects than substance type. The binary classification of the drug-type variable to (LSD vs non-LSD) might, however, fail to capture important nuances that could influence the relationship between psychedelic type and HPPD symptoms. For example, it does not account for within-group variations such as differences in the frequency and dosage of prior LSD use, which are factors that have been demonstrated to significantly impact the likelihood of experiencing perceptual disturbances (97, 98). It is also possible that an interaction between dose and substance type exists , e.g. high-dose LSD users might experience significant perceptual disturbances, while low-dose or therapeutic users might not, leading to an overall null effect when combined into a single binary category. Future research could employ multivariate analyses to understand how different variables, such as dose and frequency, interact to influence the outcomes.

Although anxiety is frequently found to be associated with greater risk of HPPD and other disturbances (35, 76), it was not found to be a significant predictor. The secondary logistic regression model has a low pseudo R-squared value of 0.015, indicating a poor fit. Further research with larger sample sizes are necessary.

### Strengths and limitations

For a general discussion of the strengths and limitations of this study’s design, see Haijen (80). We briefly summarize some of these here. Firstly, online self-surveying is vulnerable to poor control and accuracy of reporting. The present study design recorded an extensive range of details about the study cohort and their acute psychedelic experience; however, important data such as the age of initiation of psychedelics, average time periods between occasions of use, the dose and type taken during previous occasions, and the frequency of HPPD effects at baseline, before the assessed psychedelic experience, were not included. Whether individuals had previously microdosed or taken high doses of psychedelics and other substances, was also unaccounted for. Some have speculated that certain psychedelic drugs such as LSD (30), in addition to nonpsychedelic substances such as phencyclidine and cannabinoids (48), may raise the likelihood of developing HPPD symptoms compared with psilocybin. Respondents to a questionnaire on the perceptions of “hallucinogens”, tended to associate more perceptual changes to LSD than psilocybin, for example (72). As discussed earlier, characteristics of the study population, such as psychiatric history, alcohol history and other putative psychological risk-factors such as high trait anxiety should be included in future studies investigating cognition or HPPD-like effects following psychedelic use.

As participants supplied their own substances, it was not possible to control for substance purity, which may have reduced the accuracy of the results. A study examining the purity and adulteration of substances bought online and offline found the purity of 2C-B to vary significantly (99). However, in comparison to other psychoactive drugs of potential misuse such as cocaine or MDMA (99), psychedelic substances are generally less likely to be adulterated and if adulterated, tend to be tainted with other psychedelic substances (99, 100).

This study was advertised via online postings, including on social media and drug-related websites. Whilst this helped to enhance the study population size, it may have introduced sample biases. Compared with the general population, the sample may have been more educated about psychedelic harm reduction practices and more likely to use psychedelics with well-deliberated intentions, thereby creating a favorable bias toward the occurrence or reporting of positive outcomes from use of these substances. We have already determined that the sample demonstrates higher than average levels of magical ideation at baseline.

The self-selecting and self-reporting nature of the sample and study may have caused an under-estimation of iatrogenic responses to psychedelics (e.g. as discussed by Bremler et al. (49)). Additionally, large dropout rates and the potential for attrition biases renders definitive estimations of the frequency of HPPD-like symptoms difficult based on the current sample alone, although prior work in this same population has shown dropout not to be predicted by (negative) responses to the psychedelic, but rather generic predictors of attrition, such as younger age and low conscientiousness (101).

## Conclusion

Here we present evidence of reduced schizotypal or delusional thinking 4 weeks after vs. before psychedelic use, contradicting prior hypotheses and correlational data which indicated a positive relationship between these phenotypes and lifetime psychedelic use. HPPD-like effects were common, but when qualified by distress, clinically relevant cases were extremely rare. Younger age, female gender, history of a psychiatric diagnosis and higher baseline trait absorption were associated with an elevated odds of HPPD-type effects. Future work would benefit from larger sample sizes and better experimental controls in order to enable stronger inferences about causal effects and contributing variables linked to iatrogenic responses to psychedelics. We hope this study can serve as an inspiration of greater empirical scrutiny of the potential risks of psychedelics, including in later-phase multisite clinical trials that often carry an implicit incentive not to test such things—i.e. because of a fear that they may discover information that could jeopardize the goal of medical approval. We hope a steady commitment to scientific enquiry will accompany forthcoming changes in policy and access to psychedelic drugs.

## Methods

### Study design

Data were collected as part of a large-scale, prospective online survey. Only data and aspects of the design deemed relevant to this current study are discussed. For a full review of the study design, see Haijen (80).

The online survey was advertised through social media platforms and drug-related websites. Prospective participants, who had a good grasp of the English language, and who were planning to use psilocybin/magic mushrooms/truffles, LSD/1P-LSD, ayahuasca, DMT/5-MeO-DMT, salvia divinorum, mescaline, or iboga/ibogaine in the next 3 months, were invited to register for the survey. Sign up and email reminders were performed through the purpose-built website www.psychedelicsurvey.com. All data were recorded at three timepoints, except for HPPD-type effects which were only recorded at the key 4-week endpoint. The baseline timepoint was 1 week before the planned psychedelic experience. Questions regarding the acute experience, including drug dose, type, and subjective effects, were assessed 1 day after the experience date, sub-acute endpoints were at 2 and 4 weeks post experience.

### Measures

The following relevant data were collected at baseline: age, gender, education level, employment status, nationality, history of a psychiatric diagnosis, lifetime psychedelic use, previous psychedelic use, the two primary measures of delusional ideation and magical thinking, and the secondary measures of wellbeing, state and trait anxiety, and personality trait absorption.

Data relating to the acute psychedelic experience were also collected, including the drug type, drug dose, and setting.

The three primary outcome measures were assessed according to PDI (63) for delusional ideation, the MIS for magical thinking (102), and a set of self-constructed questions evaluating HPPD-type effects (SI appendix Fig. S1).

PDI (63)—The 21-item PDI is used to assess proneness to psychosis, measuring distress, preoccupation, and conviction regarding delusional ideas (63). These include questions such as, “Do you ever feel as if there is a conspiracy against you,” “Do you ever think people can communicate telepathically?,” or “Do you ever feel as if you have been chosen by God in some way?.” A sum score is calculated by totaling the number of endorsed delusional ideas. Secondary outcomes measuring the distress level, frequency of thought recurrence, and conviction in its veracity, are rated on Likert scales with values ranging from 1 to 5 for each endorsed idea. The pathological relevance of PDI items was further analyzed in this study by calculating a distress score, totaling the ratings of the question, “How distressing is this thought?.”

MIS (102)—The 15-item brief version of the validated MIS was used in the current study, where respondents rated items binarily as false or true. The scale measures the rate of aspects such as false beliefs, invalid causations, and unrealistic, magical-type ideas. Example items include, “I have felt there were messages for me in the way things were arranged, like in a store window,” “I have sometimes felt that strangers were reading my mind,” “Numbers like 13 and 7 have 0 special powers,” or “Horoscopes are right too often for it to be a coincidence.” A sum score is calculated by totaling the number of times an item was rated true.

HPPD-type symptoms scale (SI appendix Fig. S4)—The self-constructed HPPD questionnaire was comprised of three components. First, a comprehensive checkbox list enquired about the presence of nine different visual symptoms following cessation of psychedelic use, including geometric hallucinations, false perception of movement in peripheral visual fields, flashes of colors, intensified colors, trails of images of moving objects, positive afterimages, halos around objects, macropsia, and micropsia. Participants who indicated the presence of at least one effect subsequently answered the yes-no question “Do these cause significant distress or impairment in social, occupational, or other important areas of functioning?.” Any participants answering affirmatively to distress, then received a follow-up yes-no question, “These symptoms are not due to a general medical condition (e.g. anatomical lesions and infections of the brain, visual epilepsies) and are not better accounted for by another mental disorder (e.g. delirium, dementia, Schizophrenia) or hypnopompic hallucinations.”

Modified Tellegen Absorption Scale (MODTAS) (103)—The 15 category MODTAS scale measures trait absorption, exploring individual response to stimuli and imagery. Each item is rated either true or false. Items include, “Is responsive to engaging stimuli,” “Is responsive to ‘inductive’ stimuli,” “Often thinks in images,” “Can summon vivid and suggestive images,” “Has ’crossmodal’ experiences (e.g. synesthesia),” “Can become absorbed in own thoughts and imaginings,” “Can vividly reexperience the past,” “Has episodes of expanded (e.g. ESP-like) awareness,” and “Has experienced altered states of consciousness.”

### Statistical analysis

To test the association between lifetime psychedelic use and the primary measures of delusional and magical ideation, two-tailed Spearman correlations were performed. Secondary partial correlations were carried out to adjust for age as a potential confounding factor. To explore the prospective effect of the acute psychedelic experience on delusional and magical ideation, participants were further assigned into groups based on previous psychedelic use experience, in order to ensure that significant effects would not be lost due to previous psychedelic-induced changes, i.e. ceiling effects due to changes induced by prior use. To do this, participants with a lifetime psychedelic use of less than six times were assigned to a “novice” group, and those who had used more than five times were assigned to an “experienced” group. Friedman and Wilcoxon signed rank tests were used to compare the scores measured at each timepoint. Comparisons made between novice and experienced groups were performed with Mann–Whitney *U* tests. To evaluate whether changes in the primary measures were associated with changes in the secondary measures, two-tailed Pearson correlations were performed with week 4 and baseline score differences.

To investigate predictors of increased PDI and MIS after the acute psychedelic experience, a logistic regression model was performed with variables which did not exhibit collinearity based on variance inflation factors using a limit of < 10 (104). PDI and MIS outcome was binarized into groups based on whether the score did or did not increase after the acute psychedelic experience, categorized by a change score of > 0 or ≤ 0, respectively. The variables included were age, gender ( female=0, male=1), baseline personality trait absorption and frequency of psychedelic use during the last 6 months, and aspects of the acute psychedelic experience including setting (nonretreat=0, retreat=1), drug type (LSD vs. other psychedelic), drug dose, and concomitant nonpsychedelic drug use (polydrug use). Odds ratios were calculated to interpret the results. To investigate the predictors of HPPD-type symptoms, a logistic regression model was performed including the same predictor variables as above with the addition of psychiatric history (yes vs. no), again with variables exhibiting collinearity removed based on variance inflation factors using a limit of < 10 (104). The outcome was binarized into groups based on whether or not subjects reported at least one HPPD symptom. A secondary multinomial logistic regression model was performed to investigate whether any specific psychiatric diagnosis was a predictor. Corresponding correlational matrices can be accessed in the SI appendix Figs. S2, S3, S4.

Statistical analyses were performed using the NumPy, Scipy, SKlearn, Statsmod, and Pinguoin libraries in Python. A P<0.05 value was considered statistically significant. “Weak” and “moderate” correlations were determined using conventional thresholds for *r* and $r_{s}$ values “0.1-0.3,” “0.4-0.6”, respectively (105).

## Supplementary Material

pgae560_Supplementary_Data

## Data Availability

The anonymized data collected are available as open data via the Open Science Framework (OSF) online data repository.

## Preprints

This manuscript was posted on a preprint: https://doi.org/10.31234/osf.io/txzha.

## References

[1] Vollenweider FX, Vollenweider-Scherpenhuyzen MFI, Bäbler A, Vogel H, Hell D. 1998. Psilocybin induces schizophrenia-like psychosis in humans via a serotonin-2 agonist action. Neuroreport. 9(17):3897–3902. doi:10.1097/00001756-199812010-00024 9875725

[2] Carhart-Harris RL, et al 2016. The paradoxical psychological effects of lysergic acid diethylamide (LSD). Psychol Med. 46(7):1379–1390. doi:10.1017/S0033291715002901 26847689

[3] Carhart-Harris R, et al 2021. Trial of psilocybin versus escitalopram for depression. N Engl J Med. 384(15):1402–1411. doi:10.1056/NEJMoa2032994 33852780

[4] Stauffer CS, Anderson BT, Ortigo KM, Woolley J. 2021. Psilocybin-assisted group therapy and attachment: observed reduction in attachment anxiety and influences of attachment insecurity on the psilocybin experience. ACS Pharmacol Transl Sci. 4(2):526–532. doi:10.1021/acsptsci.0c00169 33860182 PMC8033604

[5] Mans K, et al 2021. Sustained, multifaceted improvements in mental well-being following psychedelic experiences in a prospective opportunity sample. Front Psychiatry. 12:1038. doi:10.3389/fpsyt.2021.647909 PMC8277190 34267683

[6] Griffiths RR, et al 2011. Psilocybin occasioned mystical-type experiences: immediate and persisting dose-related effects. Psychopharmacology (Berl). 218(4):649–665.doi:10.1007/s00213-011-2358-5 21674151 PMC3308357

[7] Schmid Y, Liechti ME. 2018. Long-lasting subjective effects of LSD in normal subjects. Psychopharmacology (Berl). 235(2):535–545. doi:10.1007/s00213-017-4733-3 28918441 PMC5813062

[8] Ly C, et al 2018. Psychedelics promote structural and functional neural plasticity. Cell Rep. 23(11):3170–3182. doi:10.1016/j.celrep.2018.05.022 29898390 PMC6082376

[9] Lyons T, Carhart-Harris RL. 2018. Increased nature relatedness and decreased authoritarian political views after psilocybin for treatment-resistant depression. J Psychopharmacol. 32(7):811–819. doi:10.1177/0269881117748902 29338538 PMC6047302

[10] Timmermann C, et al 2018. DMT models the near-death experience. Front Psychol. 9:395026. doi:10.3389/fpsyg.2018.01424 PMC6107838 30174629

[11] Teixeira PJ, et al 2022. Psychedelics and health behaviour change. J Psychopharmacol.. 36(1):12–19. doi:10.1177/02698811211008554 34053342 PMC8801670

[12] Mason NL, et al 2021. Spontaneous and deliberate creative cognition during and after psilocybin exposure. Transl Psychiatry. 11(1):209. doi:10.1038/s41398-021-01335-5 33833225 PMC8032715

[13] Kiraga MK, et al 2021. Persisting effects of ayahuasca on empathy, creative thinking, decentering, personality, and well-being. Front Pharmacol. 12:2714. doi:10.3389/fphar.2021.721537 PMC8517265 34658861

[14] Kettner H, Gandy S, Haijen ECHM, Carhart-Harris RL. 2019. From egoism to ecoism: psychedelics increase nature relatedness in a state-mediated and context-dependent manner. Int J Environ Res Public Health. 16(24):5147. doi:10.3390/ijerph16245147 31888300 PMC6949937

[15] Kettner H, et al 2021. Psychedelic communitas: intersubjective experience during psychedelic group sessions predicts enduring changes in psychological wellbeing and social connectedness. Front Pharmacol. 12:234. doi:10.3389/fphar.2021.623985 PMC8114773 33995022

[16] Studerus E, Kometer M, Hasler F, Vollenweider FX. 2011. Acute, subacute and long-term subjective effects of psilocybin in healthy humans: a pooled analysis of experimental studies. J Psychopharmacol. 25(11):1434–1452. doi:10.1177/0269881110382466 20855349

[17] Hodge AT, Sukpraprut-Braaten S, Narlesky M, Strayhan RC. 2022. The use of psilocybin in the treatment of psychiatric disorders with attention to relative safety profile: a systematic review. J Psychoactive Drugs. 55(1):40–50. doi:10.1080/02791072.2022.2044096 35225726

[18] Metzner R . 1969. A note on the treatment of LSD psychosis: a case report. Psychotherapy Theory Res Pract. 6(3):201–205. doi:10.1037/h0088751

[19] Huang HH, Bai YM. 2011. Persistent psychosis after ingestion of a single tablet of ’2C-B’. ISSN 02785846.

[20] Penn A . 2021. An episode of mania following self-reported ingestion of psilocybin mushrooms in a woman previously not diagnosed with bipolar disorder: a case report. doi:https://www.researchgate.net/publication/351285010. 10.1111/bdi.13095 33934465

[21] Palamar JJ, Le A. 2018. Trends in DMT and other tryptamine use among young adults in the United States. Am J Addict. 27(7):578–585. doi:10.1111/ajad.12803 30260086 PMC6182767

[22] Yockey RA, Vidourek RA, King KA. 2020. Trends in LSD use among US adults: 2015–2018. Drug Alcohol Depend. 212:108071. doi:10.1016/j.drugalcdep.2020.108071 32450479

[23] Yockey A, King K. 2021. Use of psilocybin (“mushrooms”) among US adults: 2015–2018. J Psychedelic Stud. 5(1):17–21. doi:10.1556/2054.2020.00159

[24] Killion B, et al 2021. LSD use in the United States: trends, correlates, and a typology of US. Drug Alcohol Depend. 223:108715. doi:10.1016/j.drugalcdep.2021.108715 33887665

[25] Schlag AK, Aday J, Salam I, Neill JC, Nutt DJ. 2022. Adverse effects of psychedelics: from anecdotes and misinformation to systematic science. J Psychopharmacol.. 36(3):258–272. doi:10.1177/02698811211069100 35107059 PMC8905125

[26] Smart RG, Storm T, Baker EF, Solursh L. 1966. A controlled study of lysergide in the treatment of alcoholism. 1. The effects on drinking behavior. Q J Stud Alcohol. 27(3):469–482. doi:10.15288/qjsa.1966.27.469 5970697

[27] Lebedev AV, et al 2021. Psychedelic drug use and schizotypy in young adults. Sci Rep. 11(1):15058. doi:10.1038/s41598-021-94421-z 34301969 PMC8302700

[28] Weiss B, Nygart V, Pommerencke LM, Carhart-Harris RL, Erritzoe D. 2021. Examining psychedelic-induced changes in social functioning and connectedness in a naturalistic online sample using the five-factor model of personality. Front Psychol. 12:5245. doi:10.3389/fpsyg.2021.749788 PMC8655335 34899488

[29] Forstmann M, Yudkin DA, Prosser AMB, Heller SM, Crockett MJ. 2020. Transformative experience and social connectedness mediate the mood-enhancing effects of psychedelic use in naturalistic settings. Proc Natl Acad Sci U S A. 117(5):2338–2346. doi:10.1073/pnas.1918477117 31964815 PMC7007572

[30] Halpern JH, Pope HG. 2003. Hallucinogen persisting perception disorder: what do we know after 50 years? https://pubmed.ncbi.nlm.nih.gov/12609692/. ISSN 03768716. 10.1016/s0376-8716(02)00306-x 12609692

[31] Cerón Tapia HR, González Guzmán MA, Córdoba Ortiz SA. 2021. Ayahuasca-induced psychosis: a case report. Rev Colomb Psiquiatr. 51 (3):236–239. doi:10.1016/j.rcp.2020.11.014 36075857

[32] Singh G, Laucis NC, Anbalagan E, Malhotra K, Gopalkrishna G. 2018. A case study of LSD induced late psychosis in a 19-year-old woman. Research Reports. 1:e1–e3. doi:10.9777/rr.2018.10334

[33] Nutting S, Bruinsma T, Anderson M, Jolly T. 2021. Psychotic and still tripping-hallucinogen persisting perception disorder and first break psychosis in an adolescent, https://pennstate.pure.elsevier.com/en/publications/psychotic-and-still-trippinghallucinogen-persisting-perception-di. ISSN 15571882.

[34] Cohen S . 1960. Lysergic acid diethylamide: side effects and complications. J Nerv Ment Dis. 130(1):30–40. https://journals.lww.com/jonmd/Fulltext/1960/01000/LYSERGIC_ACID_DIETHYLAMIDE__SIDE_EFFECTS_AND.5.aspx. 13811003 10.1097/00005053-196001000-00005

[35] Halpern JH, Lerner AG, Passie T. 2018. A review of hallucinogen persisting perception disorder (HPPD) and an exploratory study of subjects claiming symptoms of HPPD. Curr Top Behav Neurosci. 36:333–360. doi:10.1007/7854_2016_457 27822679

[36] Abraham HD, Aldridge AM. 1993. Adverse consequences of lysergic acid diethylamide. Addiction. 88(10):1327–1334. 8251869 10.1111/j.1360-0443.1993.tb02018.x

[37] Chan G, et al 2022. An age-period-cohort analysis of trends in psychedelic and ecstasy use in the Australian population. Addict Behav. 127:107216. doi:10.1016/j.addbeh.2021.107216 34979428

[38] Sandison RA, Spencer AM, Whitelaw JD. 1954. The therapeutic value of lysergic acid diethylamide in mental illness. J Ment Sci. 100(419):491–507. doi:10.1192/bjp.100.419.491 13175011

[39] Passie T, Halpern JH, Stichtenoth DO, Emrich HM, Hintzen A. 2008. The pharmacology of lysergic acid diethylamide: a review https://www.ncbi.nlm.nih.gov/pmc/articles/PMC6494066/. ISSN 17555930. 10.1111/j.1755-5949.2008.00059.x PMC6494066 19040555

[40] de Montalembert M, Coulon N, Cohen D, Bonnot O, Tordjman S. 2016. Time perception of simultaneous and sequential events in early-onset schizophrenia. Neurocase. 22(4):392–399. doi:10.1080/13554794.2016.1205098 27388526

[41] Gregorio DD, Comai S, Posa L, Gobbi G. 2016. D-lysergic acid diethylamide (LSD) as a model of psychosis: mechanism of action and pharmacology. Int J Mol Sci. 17(11):1953. doi:10.3390/ijms17111953 27886063 PMC5133947

[42] Malleson N . 1971. Acute adverse reactions to LSD in clinical and experimental use in the United Kingdom. Br J Psychiatry. 118(543):229–230. doi:10.1192/bjp.118.543.229 4995932

[43] Hartogsohn I . 2017. Constructing drug effects: a history of set and setting. Drug Sci Policy Law. 3:205032451668332. doi:10.1177/2050324516683325

[44] Uthaug MV, et al 2021. A placebo-controlled study of the effects of ayahuasca, set and setting on mental health of participants in ayahuasca group retreats. Psychopharmacology (Berl). 238(7):1899–1910. doi:10.1007/s00213-021-05817-8 33694031 PMC8233273

[45] Strickland JC, Garcia-Romeu A, Johnson MW. 2021. Set and setting: a randomized study of different musical genres in supporting psychedelic therapy. ACS Pharmacol Transl Sci. 4(2):472–478. doi:10.1021/acsptsci.0c00187 33860177 PMC8033606

[46] Carhart-Harris RL, et al 2018. Psychedelics and the essential importance of context. J Psychopharmacol. 32(7):725–731. doi:10.1177/0269881118754710 29446697

[47] Sandison RA, Whitelaw JD. 1957. Further studies in the therapeutic value of lysergic acid diethylamide in mental illness. J Ment Sci. 103(431):332–343. doi:10.1192/bjp.103.431.332 13429304

[48] Martinotti G, et al 2018. Hallucinogen persisting perception disorder: etiology, clinical features, and therapeutic perspectives. https://www.ncbi.nlm.nih.gov/pmc/articles/PMC5870365/. ISSN 20763425. 10.3390/brainsci8030047 PMC5870365 29547576

[49] Bremler R, Katati N, Shergill P, Erritzoe D, Carhart-Harris R. 2023. Focusing on the negative: cases of long-term negative psychological responses to psychedelics. https://psyarxiv.com/yzmcj/. doi:10.31234/OSF.IO/YZMCJ PMC10519946 37749109

[50] Studerus E, Gamma A, Vollenweider FX. 2010. Psychometric evaluation of the altered states of consciousness rating scale (OAV). PLoS One. 5(8):e12412. doi:10.1371/journal.pone.0012412 20824211 PMC2930851

[51] Goodwin GM, et al 2023. Single-dose psilocybin for a treatment-resistant episode of major depression: impact on patient-reported depression severity, anxiety, function, and quality of life. J Affect Disord. 327:120–127. doi:10.1016/j.jad.2023.01.108 36740140

[52] Griffiths RR, et al 2016. Psilocybin produces substantial and sustained decreases in depression and anxiety in patients with life-threatening cancer: a randomized double-blind trial. J Psychopharmacol. 30(12):1181–1197. doi:10.1177/0269881116675513 27909165 PMC5367557

[53] Grob CS, et al 2011. Pilot study of psilocybin treatment for anxiety in patients with advanced-stage cancer. Arch Gen Psychiatry. 68(1):71. doi:10.1001/archgenpsychiatry.2010.116 20819978

[54] Ross S, et al 2016. Rapid and sustained symptom reduction following psilocybin treatment for anxiety and depression in patients with life-threatening cancer: a randomized controlled trial. J Psychopharmacol. 30(12):1165–1180. doi:10.1177/0269881116675512 27909164 PMC5367551

[55] Bogenschutz MP, et al 2015. Psilocybin-assisted treatment for alcohol dependence: a proof-of-concept study. J Psychopharmacol. 29(3):289–299. doi:10.1177/0269881114565144 25586396

[56] Halpern JH, Sherwood AR, Hudson JI, Yurgelun-Todd D, Pope HG Jr. 2005. Psychological and cognitive effects of long-term peyote use among native Americans. Biol Psychiatry. 58(8):624–631. doi:10.1016/j.biopsych.2005.06.038 16271313

[57] Krebs TS, Johansen P-Ø. 2013. Psychedelics and mental health: a population study. PLoS One. 8(8):e63972. doi:10.1371/journal.pone.0063972 23976938 PMC3747247

[58] Duva SM, Silverstein SM, Spiga R. 2011. Impulsivity and risk-taking in co-occurring psychotic disorders and substance abuse. Psychiatry Res. 186(2-3):351–355. doi:10.1016/j.psychres.2010.08.014 20870294

[59] Nanda P, et al 2016. Impulsivity across the psychosis spectrum: correlates of cortical volume, suicidal history, and social and global function. Schizophr Res. 170(1):80–86. doi:10.1016/j.schres.2015.11.030 26711526

[60] Clark L, Roiser JP, Robbins TW, Sahakian BJ. 2009. Disrupted ‘reflection’ impulsivity in cannabis users but not current or former ecstasy users. J Psychopharmacol. 23(1):14–22. doi:10.1177/0269881108089587 18515464 PMC2637477

[61] Tupper KW, Wood E, Yensen R, Johnson MW. 2015. Psychedelic medicine: a re-emerging therapeutic paradigm. CMAJ. 187(14):1054–1059.doi:10.1503/cmaj.141124 26350908 PMC4592297

[62] Fonseca-Pedrero E, Paino M, Santarén-Rosell M, Lemos-Giráldez S, Muñiz J. 2012. Psychometric properties of the Peters et al Delusions Inventory 21 in college students. Compr Psychiatry. 53(6):893–899.doi:10.1016/j.comppsych.2012.01.007 22440833

[63] Peters E, Joseph S, Day S, Garety P. 2004. Measuring delusional ideation: the 21-item Peters et al. Delusions Inventory (PDI). Schizophr Bull. 30(4):1005–1022. doi:10.1093/oxfordjournals.schbul.a007116 15954204

[64] Green MJ, Williams LM, Davidson DJ. 2001. Processing of threat-related affect is delayed in delusion-prone individuals. Br J Clin Psychol. 40(2):157–165. doi:10.1348/014466501163607 11446237

[65] Rodier M, et al 2011. Healthy people with delusional ideation change their mind with conviction. Psychiatry Res. 189(3):433–439. doi:10.1016/j.psychres.2011.06.018 21763003

[66] Verdoux H, et al 1998. A survey of delusional ideation in primary-care patients. Psychol Med. 28(1):127–134. doi:10.1017/S0033291797005667 9483688

[67] Karcher NR, Slutske WS, Kerns JG, Piasecki TM, Martin NG. 2014. Sex differences in magical ideation: a community-based twin study. Personal Disord. 5(2):212–219. doi:10.1037/per0000040 24364500 PMC4065014

[68] Timmermann C, et al 2021. Psychedelics alter metaphysical beliefs. Sci Rep. 11(1):22166. doi:10.1038/s41598-021-01209-2 34815421 PMC8611059

[69] Roche SM, McConkey KM. 1990. Absorption: nature, assessment, and correlates. J Pers Soc Psychol. 59(1):91–101. doi:10.1037/0022-3514.59.1.91

[70] Brouwer A, Carhart-Harris RL. 2021. Pivotal mental states.J Psychopharmacol. 35(4):319–352.doi:10.1177/0269881120959637 33174492 PMC8054165

[71] Abraham HD . 1982. A chronic impairment of colour vision in users of LSD. Br J Psychiatry. 140(5):518–520. doi:10.1192/bjp.140.5.518 6980680

[72] Carhart-Harris RL, Nutt DJ. 2010. User perceptions of the benefits and harms of hallucinogenic drug use: a web-based questionnaire study. J Subst Use. 15(4):283–300. doi:10.3109/14659890903271624

[73] Baggott MJ, Coyle JR, Erowid E, Erowid F, Robertson LC. 2011. Abnormal visual experiences in individuals with histories of hallucinogen use: a web-based questionnaire. Drug Alcohol Depend. 114(1):61–67. doi:10.1016/j.drugalcdep.2010.09.006 21035275

[74] Davis AK, et al 2021. Effects of psilocybin-assisted therapy on major depressive disorder: a randomized clinical trial. JAMA Psychiatry. 78(5):481–489. doi:10.1001/jamapsychiatry.2020.3285 33146667 PMC7643046

[75] Glicksohn J, Barrett TR. 2003. Absorption and hallucinatory experience. Appl Cogn Psychol. 17(7):833–849. doi:10.1002/acp.913

[76] Irvine A, Luke D. 2022. Apophenia, absorption and anxiety: evidence for individual differences in positive and negative experiences of hallucinogen persisting perceptual disorder. J Psychedelic Stud. 6(2):88–103. doi:10.1556/2054.2022.00195

[77] Lerner AG, et al 2003. Clonazepam treatment of lysergic acid diethylamide-induced hallucinogen persisting perception disorder with anxiety features. Int Clin Psychopharmacol. 18(2):101–105. doi:10.1097/00004850-200303000-00007 12598822

[78] Menzies V, Taylor AG, Bourguignon C. 2008. Absorption: an individual difference to consider in mind-body interventions. J Holist Nurs. 26(4):297–302. doi:10.1177/0898010107307456 19126883 PMC2810559

[79] Kealy D, McCloskey KD, Cox DW, Ogrodniczuk JS, Joyce AS. 2019. Getting absorbed in group therapy: absorption and cohesion in integrative group treatment. Couns Psychother Res. 19(3):286–293.doi:10.1002/capr.12226

[80] Haijen ECHM, et al 2018. Predicting responses to psychedelics: a prospective study. Front Pharmacol. 9:897. doi:10.3389/fphar.2018.00897 30450045 PMC6225734

[81] Russ SL, Carhart-Harris RL, Maruyama G, Elliott MS. 2019. States and traits related to the quality and consequences of psychedelic experiences. Psychol Consciousness Theory Res Pract. 6(1):1–21. doi:10.1037/cns0000169

[82] Studerus E, Gamma A, Kometer M, Vollenweider FX. 2012. Prediction of psilocybin response in healthy volunteers. PLoS One. 7:e30800. doi:10.1371/journal.pone.0030800 22363492 PMC3281871

[83] Kessler RC, et al 2005. Lifetime prevalence and age-of-onset distributions of DSM-IV disorders in the national comorbidity survey replication. https://pubmed.ncbi.nlm.nih.gov/15939837/. ISSN 0003990X. 10.1001/archpsyc.62.6.593 15939837

[84] Arseneault L, et al 2002. Cannabis use in adolescence and risk for adult psychosis: longitudinal prospective study. Br Med J. 325(7374):1212–1213. doi:10.1136/bmj.325.7374.1212 12446537 PMC135493

[85] Grov C, Kelly BC, Parsons JT. 2009. Polydrug use among club-going young adults recruited through time-space sampling. Subst Use Misuse. 44(6):848–864. doi:10.1080/10826080802484702 19444726 PMC2683356

[86] Studerus E, Gamma A, Vollenweider FX. 2010. Psychometric evaluation of the altered states of consciousness rating scale (OAV). PLoS One. 5(8):e12412. 20824211 10.1371/journal.pone.0012412 PMC2930851

[87] Becker AM, et al 2022. Acute effects of psilocybin after escitalopram or placebo pretreatment in a randomized, double-blind, placebo-controlled, crossover study in healthy subjects. Clin Pharmacol Ther. 111(4):886–895. doi:10.1002/cpt.2487 34743319 PMC9299061

[88] Zucker I, Prendergast BJ. 2020. Sex differences in pharmacokinetics predict adverse drug reactions in women. Biol Sex Differ. 11(1):32. doi:10.1186/s13293-020-00308-5 32503637 PMC7275616

[89] Frokjaer VG, Erritzoe D, Madsen J, Paulson OB, Knudsen GM. 2009. Gender and the use of hormonal contraception in women are not associated with cerebral cortical 5-HT 2A receptor binding. Neuroscience. 163(2):640–645. doi:10.1016/j.neuroscience.2009.06.052 19559762

[90] Baker C . 2023. Obesity statistics–house of commons library, https://commonslibrary.parliament.uk/research-briefings/sn03336/.

[91] Spriggs MJ, et al 2022. Body mass index (BMI) does not predict responses to psilocybin. J Psychopharmacol. 37(1):026988112211319. doi:10.1177/02698811221131994 PMC9834321 36373934

[92] Carhart-Harris RL, et al 2018. Psilocybin with psychological support for treatment-resistant depression: six-month follow-up. Psychopharmacology (Berl). 235(2):399–408. doi:10.1007/s00213-017-4771-x 29119217 PMC5813086

[93] Pasquini L, Palhano-Fontes F, Araujo DB. 2020. Subacute effects of the psychedelic ayahuasca on the salience and default mode networks. J Psychopharmacol. 34:623–635. doi:10.1177/0269881120909409 32255395

[94] Carhart-Harris RL, et al 2016. Neural correlates of the LSD experience revealed by multimodal neuroimaging. Proc Natl Acad Sci U S A. 113:4853–4858. doi:10.1073/PNAS.1518377113 27071089 PMC4855588

[95] Bedford P, et al 2023. The effect of lysergic acid diethylamide (LSD) on whole-brain functional and effective connectivity. Neuropsychopharmacology. 48:1175–1183. doi:10.1038/s41386-023-01574-8 37185950 PMC10267115

[96] Preller KH, Vollenweider FX. 2018. Phenomenology, structure, and dynamic of psychedelic states. In: *Current topics in behavioral neurosciences*. vol. 36. Springer Verlag, p. 221–256. https://pubmed.ncbi.nlm.nih.gov/28025814/. 10.1007/7854_2016_459 28025814

[97] Hirschfeld T, Prugger J, Majić T, Schmidt TT. 2023. Dose-response relationships of lsd-induced subjective experiences in humans. Neuropsychopharmacology. 48:1602–1611. doi:10.1038/s41386-023-01588-2 37161078 PMC10516880

[98] Holze F, et al 2021. Acute dose-dependent effects of lysergic acid diethylamide in a double-blind placebo-controlled study in healthy subjects. Neuropsychopharmacology. 46:537–544. doi:10.1038/S41386-020-00883-6 33059356 PMC8027607

[99] van der Gouwe D, Brunt TM, van Laar M, van der Pol P. 2017. Purity, adulteration and price of drugs bought on-line versus off-line in the Netherlands. Addiction. 112(4):640–648. doi:10.1111/add.13720 27936283

[100] Fregonese M, et al 2021. Drug checking as strategy for harm reduction in recreational contests: evaluation of two different drug analysis methodologies. Front Psychiatry. 12:596895. doi:10.3389/fpsyt.2021.596895 33692707 PMC7938318

[101] Hübner S, Haijen E, Kaelen M, Carhart-Harris RL, Kettner H. 2021. Turn on, tune in, and drop out: predictors of attrition in a prospective observational cohort study on psychedelic use. J Med Internet Res. 23(7):e25973. doi:10.2196/25973 34319246 PMC8367105

[102] Eckblad M, Chapman L. 1983. Magical ideation as an indicator of schizotypy. J Consult Clin Psychol. 51(2):215–225. 6841765 10.1037//0022-006x.51.2.215

[103] Tellegen A, Atkinson G. 1974. Openness to absorbing and self-altering experiences (“absorption”), a trait related to hypnotic susceptibility. J Abnorm Psychol. 83(3):268–277. 4844914 10.1037/h0036681

[104] Hair RE, Black JF, Babin WC, Anderson BJ. 2013. Multivariate Data Analysis - Joseph F. Hair, William C. Black, Barry J. Babin, Rolph E. Anderson - Google Books, https://books.google.co.uk/books/about/Multivariate_Data_Analysis.html?id=VvXZnQEACAAJ&redir_esc=y.

[105] Dancey CP, Reidy J. 2007. Statistics without maths for psychology. Pearson Education.

