# Supplementary material for: Prediction of hallucinogen persisting perception disorder and thought disturbance symptoms following psychedelic use
Source: PNAS Nexus. 2025 Apr 22;4(4):pgae560. doi: 10.1093/pnasnexus/pgae560 (PMC12012689; doi:10.1093/pnasnexus/pgae560)
Supplement: pgae560_Supplementary_Data [file pgae560_supplementary_data.pdf]

Supporting Information for

Prediction of hallucinogen persisting perception disorder and thought disturbance symptoms following psychedelic use

Katie Zhou, David de Wied, Robin Carhart-Harris and Hannes Kettner

Corresponding Author name: Katie Zhou
E-mail: katie.zhou1@nhs.net

This PDF file includes:

Figs. S1 to S4
Tables S1 to S2

Variable	Coefficient	Std. error	z	*p*-value	OR [95% CI]
Age	-.01	.02	-.31	.76	.99 [.96, 1.03]
Gender	-.42	.42	-1.00	.32	.66 [.29, 1.50]
Absorption	.01	.01	.01	.49	1.01 [.98, 1.03]
LSD use[1]	.14	.45	.32	.75	.73 [.48, 2.76]
Dose	-.22	.18	-1.23	.22	1.18 [.56, 1.14]
Setting	.16	.58	.28	.28	.80 [.38,3.67]
Polydrug use[1]	.23	.42	.55	.58	1.26 [.55,2.88]
Use of psychedelic drugs[2]	-.31	.21	-1.50	.13	.73 [.48, 1.10]

Table S1. Results from the logistic regression model investigating predictors of delusional ideation. [1]During the acute experience. [2]During the last 6 months.

Variable	Coefficient	Std. error	z	*p*-value	OR [95% CI]
Depression	-.35	.39	.89	.37	< .001 [.66, 3.06]
Bipolar disorder	-.31	.53	-.59	.56	< .001 [.26, 2.06]
Schizophrenia	-18.40	> 1.00	0.00	1.00	< .001 [0.00, ∞]
Anxiety disorder	.06	.09	.64	.52	< .001 [.88, 1.27]
Substance abuse disorder	.10	.15	.69	.49	< .001 [.83, 1.49]
Alcohol dependence	-.02	.10	.10	.85	< .001 [.81, 1.19]
Personality disorder	-4.22	> 1.00	> 1.00	1.00	< .001 [.00, ∞]
Psychotic disorder	-.08	.15	.15	.60	< .001 [.68, 1.25]
ADHD	-.08	.07	.07	.28	< .001 [.81, 1.06]
OCD	-.04	.11	.11	.75	< .001 [.78, 1.20]
Eating disorder	.08	.06	.06	.21	< .001 [.96, 1.22]

Table S2. Results from the secondary multinomial logistic regression model investigating predictors of HPPD-type symptoms.

Measure	1	2	3	4	5	6	7	8
1. Age	-							
2. Gender	-.03	-						
3. Absorption	.10	-.12	-					
4. LSD use[1]	-.38	.10	-.10	-				
5. Dose	-.14	**.18**	-.14	.14	-			
6. Setting	**.25**	.01	.05	**-.35**	.02	-		
7. Polydrug use[1]	-.01	-.01	.13	.19	.03	-.12	-	
8. Use of psychedelic drugs[2]	-.13	.15	.09	.08	.08	-.14	.02	-

Fig. S1. Correlation matrix of variables in the logistic regression model investigating predictors of magical ideation (MI). [1] During the acute experience. [2] During the last 6 months.

Measure	1	2	3	4	5	6	7	8
1. Age	-							
2. Gender	-.10	-						
3. Absorption	.09	-.15	-					
4. LSD use[1]	**-.39**	**.23**	-.05	-				
5. Dose	**-.19**	.16	-.11	**.22**	-			
6. Setting	.00	.00	-.08	-.07	.04	-		
7. Polydrug use[1]	-.08	.08	.15	**.24**	.05	-.06	-	
8. Use of psychedelic drugs[2]	-.01	-.02	-.03	.03	-.02	-.11	.03	-

Fig. S2. Correlation matrix of variables in the logistic regression model investigating predictors of delusional ideation.[1] During the acute experience. [2] During the last 6 months.

**Measure**	**1**	**2**	**3**	**4**	**5**	**6**	**7**	**8**	**9**
1. Age	-								
2. Gender	-.01	-							
3. Absorption	.05	-.07	-						
4. Psychiatric history	-.04	-.04	**.24**	-					
5. LSD use[1]	**-.37**	.10	-.07	-.10	-				
6. Dose	-.14	**.18**	-.12	.12	.17	-			
7. Setting	**.22**	.05	.05	.09	**-.33**	-.10	-		
8. Polydrug use[1]	-.02	-.01	.16	.01	**.21**	.04	-.10	-	
9. Use of psychedelic drugs[2]	-.10	.13	.11	.00	.07	.11	-.13	.05	-

Fig. S3. Correlation matrix of variables in the logistic regression model investigating predictors of HPPD-type symptoms. [1] During the acute experience. [2] During the last 6 months.

Item 1

	Do you re-experience one or more of the following? *(check box)*
Geometric Hallucinations	
False Perceptions Of Movement In Peripheral Visual Fields	
Flashes Of Colours	
Intensified Colours	
Trails Of Images Of Moving Objects	
Positive After-images	
Halos Around Objects	
Macropsia (Objects Appear Larger Than They Normally Are)	
Micropsia (Objects Appear Smaller Than They Normally Are)	
None Of The Above	
Halos Around Objects	

Item 2

Do these cause significant distress or impairment in social, occupational, or other important areas of functioning?	Yes / No

Item 3

These symptoms are not due to a general medical condition (e.g. anatomical lesions and infections of the brain, visual epilepsies) and are not better accounted for by another mental disorder (e.g. delirium, dementia, Schizophrenia) or hypnopompic hallucinations	Yes / No

Fig. S4. Self-constructed, 3-item HPPD-type symptoms scale.
